# A duplex probe-directed recombinase amplification assay for detection of single nucleotide polymorphisms on 8q24 associated with prostate cancer

**DOI:** 10.1590/1414-431X20209549

**Published:** 2020-11-27

**Authors:** Qingxia Duan, Xinna Li, Xiaozhou He, Xinxin Shen, Yu Cao, Ruiqing Zhang, Xueding Bai, Jinyan Zhang, Xuejun Ma

**Affiliations:** 1 Hebei Medical University, Shijiazhuang, Hebei, China; 2 NHC Key Laboratory of Medical Virology and Viral Diseases, National Institute for Viral Disease Control and Prevention, Chinese Center for Disease Control and Prevention, Changping District, Beijing, China; 3 Hebei Medical University Fourth Affiliated Hospital, Shijiazhuang, Hebei, China; 4 Yangzhou Center for Disease Control and Prevention, Yangzhou, Jiangsu, China

**Keywords:** SNP, Genotyping, Duplex-PDRA, Prostate cancer

## Abstract

Single nucleotide polymorphisms (SNPs) have important application value in the research of population genetics, hereditary diseases, tumors, and drug development. Conventional methods for detecting SNPs are typically based on PCR or DNA sequencing, which is time-consuming, costly, and requires complex instrumentation. In this study, we present a duplex probe-directed recombinase amplification (duplex-PDRA) assay that can perform real-time detection of two SNPs (rs6983267 and rs1447295) in four reactions in two tubes at 39°C within 30 min. The sensitivity of duplex-PDRA was 2×10^3^-10^4^ copies per reaction and no cross-reactivity was observed. A total of 382 clinical samples (179 prostate cancer patients and 203 controls) from northern China were collected and tested by duplex-PDRA assay and direct sequencing. The genotyping results were completely identical. In addition, the association analysis of two SNPs with prostate cancer risk and bone metastasis was conducted. We found that the TT genotype of rs6983267 (OR: 0.42; 95%CI: 0.23-0.78; P=0.005) decreased the risk of prostate cancer, while the CA genotype of rs1447295 (OR: 1.89; 95%CI: 1.20-2.96; P=0.005) increased the risk of prostate cancer. However, no association between the two SNPs (rs6983267 and rs1447295) and bone metastasis in prostate cancer was found in this study (P>0.05). In conclusion, the duplex-PDRA assay is an effective method for the simultaneous detection of two SNPs and shows great potential for widespread use in research and clinical settings.

## Introduction

Single nucleotide polymorphism (SNP) is one of the most common forms of polymorphisms in the genome. SNPs can occur in widespread regions, such as coding regions, border exons/introns, introns, and promoter regions, and the structure, function, and stability of the encoded mRNA and protein can be altered. Thus, in addition to the response to treatment, individual gene mutations as a result of SNPs are associated with an individual's susceptibility to a variety of hereditary diseases. Therefore, SNP testing plays an important role in the early diagnosis, prevention, and treatment of certain diseases (1,2). In 2006, Amundadottir et al. found in Iceland, Sweden, and other populations that multiple SNPs in the chromosome 8q24 region are closely related to the risk of prostate cancer (PCa) by a large genome-wide association study (GWAS) (3). This correlation has been followed and confirmed in European Americans, African Americans and native Hawaiians, Spain, the Netherlands, Japan, and other peoples (4–11). China has a few studies on the correlation between 8q24 region and PCa (12,13), but the results are not completely consistent. In addition, PCa is prone to bone metastasis (14). There is no relevant report on whether PCa bone metastasis is related to SNPs located on 8q24.

Currently, there are various methods for SNPs typing such as TaqMan real-time PCR (15), amplification of refractory mutation system PCR (ARMS-PCR) (16), restriction fragment length polymorphism (RFLP) analysis (17), direct DNA sequencing (18), denatured high performance liquid chromatography (DHPLC) (19), and gene chip technology (20). Recently, isothermal amplification technology has been increasingly used due to its advantages of time-saving, cost-effectiveness, and being easy to use. Most importantly, isothermal amplification eliminates the need for expensive thermal cyclers used in thermal cycling procedures (21). These isothermal amplification techniques include loop-mediated amplification (LAMP), nucleic acid sequence-based amplification (NASBA), smart-amplification process version 2 (SMAP2), helicase-dependent amplification (HDA), and recombinase polymerase amplification (RPA), etc. Some studies have also applied these techniques to SNP detection (22-28). However, each of these methods has its own shortcomings and limits its application in SNP assay. For example, the primer design of LAMP and SMAP2 is too complex, and LAMP requires 4-6 primers and even more primers for multiple detection. NASBA is not suitable for DNA analysis and HDA has complex buffer optimization.

A novel isothermal amplification technique - recombinant aided amplification (RAA) - was originally designed to rapidly amplify target sequences at 37-42°C in less than 30 min. In our previous report, RAA was modified and named as probe-directed recombinase amplification (PDRA) for a single SNP detection (*MTHFR* A1298C) (29). In order to further save time, money, and be more efficient, in the present study, we aimed to establish a duplex real-time fluorescent PDRA method for simultaneous detection of two SNP sites (rs6983267 and rs1447295) located on 8q24, which are considered to be highly correlated with the susceptibility to PCa (3–5 [Bibr B06] [Bibr B07] [Bibr B08] [Bibr B09]).

The clinical samples from a case-control group were used to evaluate application of duplex PDRA and the association between 8q24 risk alleles (rs6983267 and rs1447295) and PCa risk and bone metastasis in northern Chinese.

## Material and Methods

### Clinical samples collection

A total of 179 patients with PCa (33 bone metastases and 146 without bone metastasis) and 203 normal subjects (control group) were enrolled in this study. All cases were diagnosed as PCa and confirmed by histology at the Department of Urology, The Fourth Hospital of Hebei Medical University between December 1, 2018 and October 1, 2019. The age-matched control group were local residents who participated in a medical examination. The control group had a negative rectal examination, low PSA levels (less than 4.0 ng/mL), and were without a family history of PCa. A total volume of 2 mL EDTA blood samples or 1 mL blood clot were collected and stored at -80°C. In this study, we collected a total of 308 EDTA blood samples (140 from the PCa group and 168 from the control group) and 74 blood clot samples (39 from the PCa group and 35 from the control group).

The study was conducted with the approval of the Institutional Review Board of National Institute for Viral Disease Control and Prevention, Center for Disease Control and Prevention of China. Written informed consent was obtained from all subjects enrolled in the study.

### DNA extraction

Genomic DNA extraction from 500 μL EDTA blood was performed by the TIANamp Blood DNA kit (Tiangen, China). The genomic DNA in the blood clot specimen was extracted using the TIANamp Blood Clot DNA kit (Tiangen, China). All of the DNA extractions were performed according to manufacturer instructions. The extracted DNA was eluted in 50 μL of elution buffer and stored at -80°C until examination. The extracted genomic DNA were quantified using a Qubit^®^ dsDNA BR Assay kits (Life Technologies Invitrogen, USA), and the final concentration ranged from 6.56 to 73.4 ng/μL.

### Duplex-PDRA mechanism and design of primers and probes

Our previously reported PDRA assay (29) for a single SNP contains two separate reactions, which is different from real-time RAA assays. Briefly, there is only one probe (46-52 bp) and one primer (30-35 bp) per reaction, and the probe itself can be used as another primer. A cleavage site (THF) is added inside the probe for exo-cleavage, and the position of the mutation site is designed to be located just before the cleavage site. On both sides of the THF site, a fluorescent group and a corresponding quenching group are labeled. The fluorophore are FAM and HEX, the quenching group is BHQ1, and the number of bases between the fluorophore and the quenching group is less than or equal to 5. The 5' end is not less than 30 bp from the THF site, and the 3' end is not less than 15 bp from the THF site. A modifying group that prevents the polymerase from extending or amplifying is marked at the 3’ end. Cleavage occurs only when the probe is bound to the template DNA, then the fluorophore is separated from the quenching group to release the fluorescence. Fluorescent signals can be read in real time with a portable, real-time fluorescence scanner.

For one SNP of duplex-PDRA assay, the primers for 2 alleles are identical, and 2 probes corresponding to 2 alleles differ in one base at the SNP position. Duplex-PDRA requires a total of 4 probes and 4 primers in 4 reactions. Four reactions are divided into 2 tubes (2 reaction units), and each reaction unit contains 2 reactions corresponding to 1 allele of 2 SNPs. Two probes in the same tube are labeled with different fluorescence, thus one tube can achieve the detection of 2 alleles corresponding to 2 SNPs, and two tubes can achieve the genotyping of 2 SNPs. For rs6983267 and rs1447295 in this study, the design of the two reaction units is shown below. The first reaction unit (tube 1) tube includes the G reaction of rs6983267 and the C reaction of rs1447295, and the second reaction unit (tube 2) includes the T reaction of rs6983267 and the A reaction of rs1447295.

All oligonucleotides (primers and probes) for the duplex-PDRA assay are shown in Table 1. rs6983267 G probe and rs1447295 A probe were labelled with the FAM fluorophore and rs6983267 T probe and rs1447295 C probe were labelled with the HEX fluorophore. All oligonucleotides were synthesized and purified by Sangon Biotech (China).

**Table 1:** Primers and probes of SNPs for the duplex probe-directed recombinase amplification assay.

Primers	Sequences (5' to 3')
rs6983267-F^*^	AATTGCTTAACCTCTTCCTATCTCAGCTCCCTA
rs6983267-G-P^†#^	ATAAAAATTCTTTGTACTTTTCTCAG[6-FAM-dT]GC[THF][BHQ1-dT]TTCATCTGCTGAGCT[C3-Spacer]
rs6983267-T-P^‡^**	ATAAAAATTCTTTGTACTTTTCTCAG[HEX-dT]GA[THF][BHQ1-dT]TTCATCTGCTGAGCT[C3-Spacer]
rs1447295-F^§^	AGTTGCACGCCAGACACTATACTAGATGATGGG
rs1447295-C-P^||^**	AAGGGGTTCCTGTTGCTTTTTTTCCATAGCA[HEX-dT]G[THF][BHQ1-dT]TTACATACCTCC[C3-Spacer]
rs1447295-A-P^¶#^	AAGGGGTTCCTGTTGCTTTTTTTCCATAGCA[6-FAM-dT]T[THF][BHQ1-dT]TTACATACCTCC[C3-Spacer]

Rs6983267 forward primer for both G and T reactions. ^†^Rs6983267 G probe. ^‡^Rs6983267 T probe. ^§^Rs1447295 forward primer for both C and A reactions. ^||^Rs1447295 C probe. ^¶^Rs1447295 A probe. ^#^Probe modifications: 6-FAM: 6-carboxyfluorescein; THF: tetrahydrofuran; BHQ1: black hole quencher 1; C_3_-Spacer: 3′ phosphate blocker. **Probe modifications: HEX: 5-hexachlorofuorescein THF: tetrahydrofuran; BHQ1: black hole quencher 1; C_3_-Spacer: 3′ phosphate blocker.

### Reaction conditions of duplex-PDRA assay

Duplex-PDRA assay was performed in a 50-μL reaction volume using RAA Exo kits (Qitian, China) according to the manufacturer's instructions. The optimized reaction system of the first reaction unit was as follows: 25 μL of rehydration buffer, 16.8 μL of DNase-free water, 1.2 μL of rs6983267 forward primer (10 μM), 0.5 μL of rs6983267 G probe (10 μM), 1.4 μL of rs1447295 forward primer (10 μM), 0.6 μL of rs1447295 C probe (10 μM), 2.5 μL of magnesium acetate (280 mΜ), and 2 μL of genomic DNA. The optimized second reaction unit included 25 μL of rehydration buffer, 14.6 μL of DNase-free water, 1.6 μL of rs6983267 forward primer (10 μM), 0.8 μL of rs6983267 T probe (10 μM), 2.1 μL of rs1447295 forward primer (10 μM), 1.4 μL of rs1447295 A probe (10 μM), 2.5 μL of magnesium acetate (280 mΜ), and 2 μL of genomic DNA. After adding the reaction mixture to a 0.2-mL freeze-dried reaction tube with dried enzyme pellet, the tube was then moved into the QT-B6100 (Qitian) to shake and mix for 4 min and then transferred to the QT-F1620 (Qitian) with FAM and HEX fluorescent detection channels at 39°C for 30 min. The results of two fluorescent channels were observed in real time. Each run included a negative control (nuclease-free water).

### Sensitivity and cross-reactivity of duplex-PDRA assay

Ten-serial dilutions of plasmid templates (10^5^ to 10^1^) were used to access the sensitivity of duplex-PDRA in this study. The plasmids harboring rs6983267 GG and rs1447295 CC were constructed and added to the first reaction unit, while plasmids containing rs6983267 TT and rs1447295 AA were constructed and added to the second reaction unit. For specificity analysis, the rs6983267 G reaction and rs1447295 C reaction in the first reaction unit were evaluated using the rs6983267 TT and the rs1447295 AA plasmids as templates, while the specificity of the rs6983267 T reaction and the rs1447295 A reaction in the second reaction unit was tested by adding the rs6983267 GG and rs1447295 CC plasmids as templates.

### PDRA assay of clinical samples and interpretation of the PDRA results

A total of 382 samples were typed by the duplex-PDRA assay under the above reaction conditions. Two SNPs of each sample were typed by combining the results of the two reaction units. Each reaction unit corresponded to 1 allele of 2 SNPs. Taking G and T reactions of rs6983267 as an example, if only one reaction unit is positive, the genotype is considered to be homozygote (GG or TT). If both reaction units are positive, the genotype is judged as heterozygote (GT).

### Direct sequencing of clinical samples

The rs6983267 and rs1447295 polymorphisms in 382 clinical samples were also detected by PCR using the QIAGEN Multiplex PCR Kit (Qiagen, Germany). The PCR product was separated and identified by gel electrophoresis and then it was subjected to direct DNA sequencing by Sanger sequencing method (Sangon Biotech). Table 2 shows the primers of rs6983267 (G/T) and rs1447295 (C/A). The 25-μL PCR reaction system contained 12.5 μL 2x QIAGEN Multiplex PCR Master mix, 8.5 μL of DNase-free water, 1 μL of each primer (10 μM), and 2 μL of DNA template. The reaction procedures were as follows: 95°C pre-denaturation for 15 min; 94°C denaturation for 30 s, 57°C annealing for 90 s, 72°C extension for 90 s, for 40 cycles; 4°C final extension for 10 min.

**Table 2:** Primers for PCR and direct sequencing.

Primers	Sequences (5' to 3')	Size (bp)
rs6983267-PCR-F	AAACCTGATTTCCCTTCCAGCTCC	463
rs6983267-PCR-R	TTGAGAAACGAGAACAGTTGTGG	
rs1447295-PCR-F	TCTCCATGAGTCTCCTTTGC	451
rs1447295-PCR-R	TCTTATCCAGCTAATGACTTCC	

## Results

### Sensitivity and cross-reactivity of duplex-PDRA assay

By using recombinant plasmids as templates, the sensitivities for each reaction of duplex-PDRA were as follows: rs6983267 G reaction and rs1447295 A reaction was 2×10^4^ copies per reaction, while rs6983267 T reaction and rs1447295 C reaction was 2×10^3^ copies per reaction. The specificity results of duplex-PDRA showed that no cross-reactivity of duplex-PDRA assays was observed when up to 10^5^ copies of recombinant plasmids were used as input templates per reaction (data not shown).

### Duplex-PDRA results of clinical samples and statistical analysis

**Figure 1:**
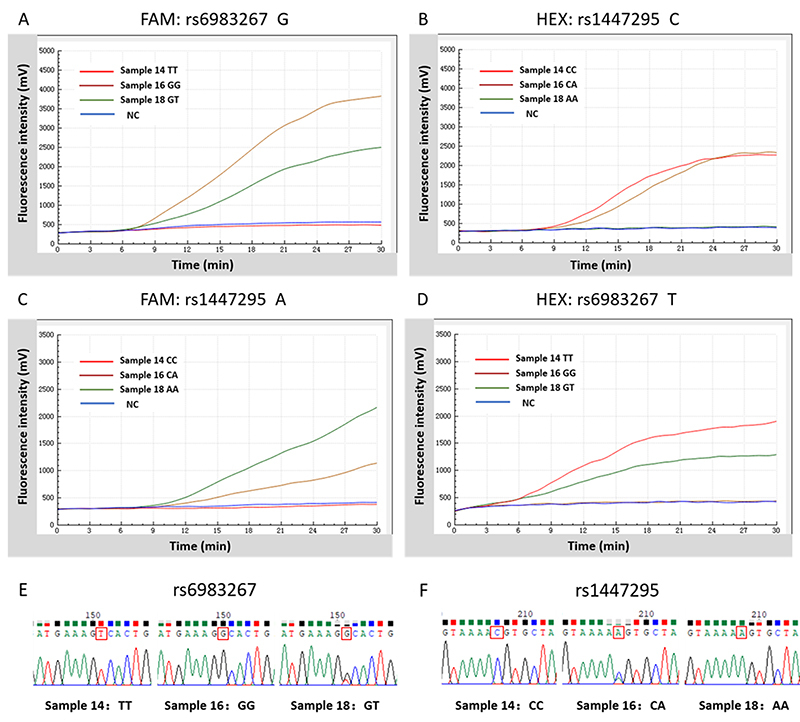
Comparison of results between three samples with different genotypes by duplex probe-directed recombinase amplification and direct sequencing.

To assess the feasibility of the duplex-PDRA assay, a total of 382 samples were tested by duplex-PDRA assay and direct DNA sequencing concurrently. The genotypes obtained from the duplex-PDRA assay were identical to the direct sequencing. Taking three samples with different genotyping results as an example, the genotyping results of these three samples were typed by duplex-PDRA assays and direct DNA sequencing method as shown in Figure 1.

Hardy-Weinberg equilibrium (HWE) analysis was performed on each SNP separately among subjects by Pearson's chi-squared test. SPSS Statistics, version 21 (IBM Corporation, USA) was used to perform the statistical analysis, and each risk allele was assessed by odds ratio (OR) and 95% confidence interval (95%CI), and the significance level was set at 0.05.

The genotype distribution for each SNP was consistent with HWE (rs6983267, χ^2^, 1.36, P=0.507; rs1447295, χ^2^, 0.06, P=0.972), indicating that the population had a representative group. Table 3 shows the distributions of the genotypes and alleles of the rs6983267 and rs1447295 polymorphisms in the PCa and control groups. In terms of genotype of rs6983267, compared with the wild genotype GG as a reference, the TT genotype (OR: 0.42; 95%CI: 0.23-0.78; P=0.005) and GT+TT genotypes (OR: 0.53; 95%CI: 0.32-0.89; P=0.016) were associated with decreased risk of PCa. In terms of genotype of rs1447295, compared with the wild genotype CC as a reference, the CA genotype (OR: 1.89; 95%CI: 1.20-2.96; P=0.005) and the CA+AA genotypes (OR: 1.95; 95%CI: 1.27-3.00; P=0.002) significantly increased the risk of PCa. In allele-wise analyses, the T allele of rs6983267 (OR: 0.68; 95%CI: 0.51-0.91; P=0.008) decreased the risk of PCa. However, the A allele of rs1447295 (OR: 1.78; 95%CI: 1.23-2.59; P=0.002) seems to be potentially associated with an increased risk of PCa.

**Table 3:** Genotypic and allelic frequencies of rs6983267 and rs1447295 polymorphisms in the PCa group (prostate cancer) and control group.

Genotype	PCa (N=179) (n, %)	Control (N=203) (n, %)	OR	95%CI	P
rs6983267					
TT	39 (21.8)	63 (31.0)	0.42	0.23-0.78	0.005
GT	96 (53.6)	110 (54.2)	0.56	0.35-1.0	0.058
GG	44 (24.6)	30 (14.8)	Reference		
GT+TT	135 (75.4)	173 (85.2)	0.53	0.32-0.89	0.016
rs1447295					
AA	9 (5.0)	5 (2.5)	2.55	0.83-7.82	0.092
CA	64 (35.8)	48 (23.6)	1.89	1.20-2.96	0.005
CC	106 (59.2)	150 (73.9)	Reference		
CA+AA	73 (40.8)	53 (26.1)	1.95	1.27-3.00	0.002

Allele	PCa (2N=358) (n, %)	Control (2N=406) (n, %)	OR	95%CI	P
rs6983267					
T	174 (48.6)	236 (58.1)	0.68	0.51-0.91	0.008
G	184 (51.4)	170 (41.9)	Reference		
rs1447295					
A	82 (22.9)	58 (14.3)	1.78	1.23-2.59	0.002
C	276 (77.1)	348 (85.7)	Reference		

Chi-squared test was used for statistical analyses.

Table 4 shows the distributions of the genotypes and alleles of the rs6983267 and rs1447295 polymorphisms in the PCa group. In this study, we found no significant difference in the genotyping distribution between rs6983267 and rs1447295 in patients with and without bone metastases in the PCa group (P>0.05).

**Table 4:** Genotypic and allelic frequencies of rs6983267 and rs1447295 polymorphisms in the PCa group.

Genotype	Bone metastases (N=33) (n, %)	No bone metastases (N=146) (n, %)	OR	95%CI	P
rs6983267					
TT	7 (21.2)	32 (21.9)	0.74	0.25-2.19	0.590
GT	16 (48.5)	80 (54.8)	0.68	0.28-1.65	0.392
GG	10 (30.3)	34 (23.3)	Reference		
GT+TT	23 (69.7)	112 (76.7)	0.70	0.30-1.61	0.398
rs1447295					
AA	7 (21.2)	7 (4.8)	0.98	0.19-5.01	0.977
CA	16 (48.5)	57 (39.0)	0.42	0.17-1.04	0.056
CC	10 (30.3)	82 (56.2)	Reference		
CA+AA	23 (69.7)	64 (43.8)	0.48	0.21-1.11	0.080

Allele	Bone metastases (2N=66) (n, %)	No bone metastases (2N=292) (n, %)	OR	95%CI	P
rs6983267					
T	30 (45.5)	144 (49.3)	0.86	0.50-1.46	0.571
G	36 (54.5)	148 (50.7)	Reference		
rs1447295					
A	11 (16.7)	71 (24.3)	0.62	0.31-1.25	0.182
C	55 (83.3)	221 (75.7)	Reference		

Chi-squared test was used for statistical analyses.

## Discussion

The duplex-PDRA assay for dual detection of SNPs was established in this study on the basis of previous PDRA study, in which the classification of *MTHFR* A1298C was successfully performed in 45 min at 39°C (29). PDRA technique utilizes an oligonucleotide probe to fully match the template strand under the action of a recombinase, a single-stranded binding protein, and a DNA polymerase to initiate amplification, and the mismatched probe initiates a later amplification or non-expansion. This strategy results in a significant difference in amplification efficiency, thereby making typing the SNP possible.

In this study, a similar strategy was adopted to test whether the PDRA method can be used to implement the concept of two-fold or even multiple detection of SNPs. At the beginning of this study, we tried to put the two reactions of one SNP in the same tube, but the result was not satisfactory, because effective amplification could not be achieved, which may be due to the interaction of the two probes that differ only by one base. Then, we put one reaction for each of the two SNPs in the same tube, so that the two tubes included four reactions, and the classification of the two SNPs was achieved.

As each tube still had two reactions, the optimization of reaction was needed. Because different target genes have unequal reaction rates when tested separately by single PDRA, it is inevitable that there will be competition in the same tube, and genes with a faster amplification rate will outperform the slower one. In order to equalize the reaction rates of the two reactions in the same tube, it is necessary to optimize the reaction conditions by adjusting the concentrations of the primers and probes. For each reaction in the same tube, 2.1 μL of primer (10 μM) and 1.4 μL of probe (10 μM) were added as the starting concentrations, and adjusted according to the result, and finally the duplex-PDRA reaction conditions were determined.

The duplex-PDRA assay includes several advantages: i) the entire assay is based on RAA, which is capable of genotyping real samples in an isothermal manner; ii) the assay is a dual test: the amplification of two different DNA targets in the same tube has the advantage to gain more information from one sample and thus reduce time and costs, and, as the number of machine detection channels increases, it is also possible to achieve multiple SNPs detection; iii) the method is simple and easy to operate, eliminating the need to open the lid and eliminating the risk of contamination; iv) the sensitivity and specificity of this method is comparable to direct sequencing, supporting its use in the clinical setting; and v) probes are sensitive enough to distinguish single-base mutations, so this strategy has a potential impact on detecting other forms of genetic variation, such as missing, insertion, and repetition.

Based on the duplex-PDRA assay results, an association study was conducted on whether rs6983267 and rs1447295 were linked to PCa patients in Hebei Province, China. The results showed that rs6983267 and rs1447295 may be closely related to PCa, the TT genotype of rs6983267 and the T allele decreased the risk of PCa, the CA genotype of rs1447295 and the A allele significantly increased the risk of PCa, which is consistent with previous research results in some countries or regions (5,10). It also suggested that the PCa risk loci identified in European populations by GWAS are associated with PCa risk in Hebei Province, Northern China, a low-risk PCa population. In the further study of whether bone metastases in the PCa group were also associated with rs6983267 and rs1447295, we found no association. However, the sample size was too small, and future studies on larger samples will be needed in the Chinese population to confirm these findings and explore the role of these risk loci in the etiology of PCa.

In the past, the prevalence of PCa in China has been low, but with the aging of the population, changes in lifestyle and eating habits, the application of digital rectal examination, PSA screening, and magnetic resonance imaging, the incidence rate has increased year by year (30). More seriously, for men diagnosed with PCa during 2010-2014, the five-year survival was approaching 100% in the US, while the survival in China was only 69% (31). The main reason for this gap is that there are few early cases of cancer in China, and the rate of early diagnosis is low. When cancer is discovered, it is already in the middle and late stages. In addition, patients with PCa suffer from pain. Bone metastasis is a major complication of patients with advanced PCa, which can cause pain, pathological fractures, spinal cord compression, and limited mobility (14). Therefore, early PCa prevention and screening of susceptible populations will not only help early detection and treatment, but also help improve patients' quality of life and improve long-term prognosis.

To our knowledge, the duplex-PDRA isothermal amplification method established in this study is the first report of RAA for the detection of 2 SNPs and the first report of RAA for detecting tumor susceptibility gene SNPs. This study provided a convenient, time-saving, cost-saving, and high-accuracy method for the early screening of PCa-associated SNPs such as rs6983267 and rs1447295.
